# Anti-galactocerebroside Antibody-Associated Bickerstaff’s Brainstem Encephalitis With Dysautonomia: A Case Report and a Review of Associated Central Nervous System Diseases

**DOI:** 10.7759/cureus.69587

**Published:** 2024-09-17

**Authors:** Masato Okitsu, Keizo Sugaya, Kazushi Takahashi

**Affiliations:** 1 Department of Neurology, Tokyo Metropolitan Neurological Hospital, Tokyo, JPN

**Keywords:** anti-galactocerebroside (gal-c) antibody, auditory brainstem response (abr), bickerstaff's brainstem encephalitis (bbe), guillain-barré syndrome (gbs), paralytic ileus

## Abstract

A 68-year-old man developed diplopia, unsteady walking, and bladder and bowel dysfunction followed by consciousness disturbance within four weeks. On physical examination, consciousness disturbance, bilateral ptosis, ophthalmoplegia, disappearing of doll's eye phenomenon, dysarthria, and diminished deep tendon reflexes were observed. Cerebrospinal fluid (CSF) examination showed oligoclonal bands. Autoantibody to galactocerebroside (Gal-C) was only positive in the serum. Blink reflex and auditory brainstem response showed abnormal findings although brain MRI and peripheral nerve conduction study were negative. The diagnosis of Bickerstaff's brainstem encephalitis (BBE) associated with anti-Gal-C antibody was made. Methylprednisolone (mPSL) pulse therapy was administered but it was not effective. The patient developed a paralytic ileus with complications and required artificial ventilation. Intravenous immunoglobulin (IVIG) was administered, and he was weaned from the ventilator two weeks later. His symptoms slowly improved, and he was discharged after four months. Anti-Gal-C antibody causes a variety of central nervous system (CNS) diseases, including brainstem encephalitis, in all ages but not many cases have been accumulated. Although reports on anti-Gal-C antibody-associated BBE are scarce, the clinical presentation of this case clearly differed from that of classic BBE.

## Introduction

Bickerstaff brainstem encephalitis (BBE) is a rare immunological disease that can present with a variety of symptoms, including motor and sensory disturbances and dysautonomia in addition to the clinical triad of ophthalmoplegia, ataxia, and consciousness disturbance [1]. Most cases develop after an antecedent infection, and approximately two-thirds of the cases are positive for serum anti-GQ1b IgG antibody among anti-ganglioside antibodies [2]. Some cases are positive for other antibodies, such as anti-GM1, GD1a, and GalNAc-GD1a IgG antibodies [3]. Treatment includes administration of intravenous immunoglobulin (IVIG) and plasma exchange, and the prognosis is generally good [4].

Here, we report a case of an older patient with anti-galactocerebroside (Gal-C) antibody-associated BBE without any episode of prior infection who presented with impaired consciousness, ataxia, and ophthalmoplegia with autonomic dysfunctions. The patient required ventilatory management and demonstrated partial response to IVIG. In relation to anti-Gal-C antibody-associated BBE, clinicians should be aware of the variation in clinical presentation from that of classic BBE and the possibility of resistance to immunotherapy and systemic complications.

## Case presentation

A 68-year-old man developed diplopia, unsteady walking, and defecation disorder four weeks before admission to our hospital. His previous physician mentioned dysarthria and ataxia of the extremities although head MRI showed no abnormality. Two weeks later, the patient developed difficulty opening the mouth and right ptosis appeared. On the day before admission to our hospital, the patient’s wife noticed a disturbance in his consciousness and they visited the emergency room of another hospital. The medical team confirmed urinary retention and placed a urethral catheter. Suspecting encephalitis, the patient was transferred to our hospital the next day. He had a history of mild untreated hypertension and was not taking any medications or supplements. He had a habit of consuming 2 liters of beer daily, although he maintained a balanced diet.

Vital signs on admission were normal; pulse was 70/min, blood pressure was 132/77 mmHg, and body temperature was 36.7°C except for the impaired Glasgow coma scale, E3V4M5. On cranial nerve examination, the pupils were 2.5 mm and the light reflex was maintained. Bilateral eye movements were strongly restricted both vertically and horizontally, and gaze-directed horizontal nystagmus was observed in the left eye while gazing to the right. Additionally, bilateral ptosis was predominant in the left and the loss of the doll's eye phenomenon was observed. He also exhibited dysarthria although we could not clearly distinguish whether his dysarthria was due to facial paralysis, ataxia, or a mixture of both. The deep tendon reflex of the limbs was hyporeflexia. Although he could not follow our orders sufficiently, the manual muscle test score of his limbs was at least four. The main results of laboratory tests are shown in Table 1.

**Table 1:** The results of laboratory tests. CK, creatine kinase; ALT, alanine aminotransferase; AST, aspartate aminotransferase; LDH, lactate dehydrogenase; HbA1c, hemoglobin A1C; CRP, C-reactive protein; RF, rheumatoid factor; ANA, antinuclear antibody; sIL-2r, soluble interleukin-2 receptor; ACE, angiotensin converting enzyme; IGRA, interferon-gamma release assay; CF, complement fixation; CSF, cerebrospinal fluid

Laboratory test	Patient value	Reference value	Units
Blood test			
Leukocytes (WBC)	9100	4000-8500	µL
Hemoglobin	14.2	13.7-16.8	g/dL
Hematocrit	39	40.7-50.1	%
Platelets	26.6	15.8-34.8	10^4^µL
Total protein	6.3	6.6-8.1	g/dL
Albumin	4.0	4.1-5.1	g/dL
Urea, plasma (BUN)	7.3	8.0-20.0	mg/dL
Creatinine	0.48	0.70-1.10	mg/dL
Anmonia (NH_3_)	32	12-66	µg/dL
Total bilirubin	1.0	0.4-1.5	mg/dL
Sodium	137	138-145	mEq/L
Potassium	4.1	3.6-4.8	mEq/L
CK	45	59-248	U/L
ALT	14	10-42	U/L
AST	18	13-30	U/L
LDH	192	124-222	U/L
Glucose	83	73-109	mg/dL
HbA1c	6.6	4.9-6.0	%
CRP	1.77	0.00-0.30	mg/dL
RF	<5	0-15	IU/mL
ANA	<1:40	0-1:40	
IgG	846	861-1747	mg/dL
IgA	187	93-393	mg/dL
IgM	155	33-183	mg/dL
sIL-2r	324	122-496	U/mL
ACE	6.6	7.0-25.0	IU/L
IGRA	-	-	
*Mycoplasma pneumoniae* antibody: CF method	1:4	<1:64	
Anti-GM1 antibody IgM	-	-	NA
IgG	-	-	NA
Anti-GM2 antibody IgM	-	-	NA
IgG	-	-	NA
Anti-GD1a antibody IgM	-	-	NA
IgG	-	-	NA
Anti-GT1b antibody IgM	-	-	NA
IgG	-	-	NA
Anti-GQ1b antibody IgM	-	-	NA
IgG	-	-	NA
Anti-Gal-C antibody IgM	-	-	NA
IgG	++	-	NA
CSF test			
Cell count	2	0-5	µL
Glucose	86	48-83	mg/dL
Protein	31	15-45	mg/dL
Myelin basic protein	<31.2	0-102	pg/mL
IgG index	0.5	<0.7	
OCBs	+	-	NA

**Figure 1:**
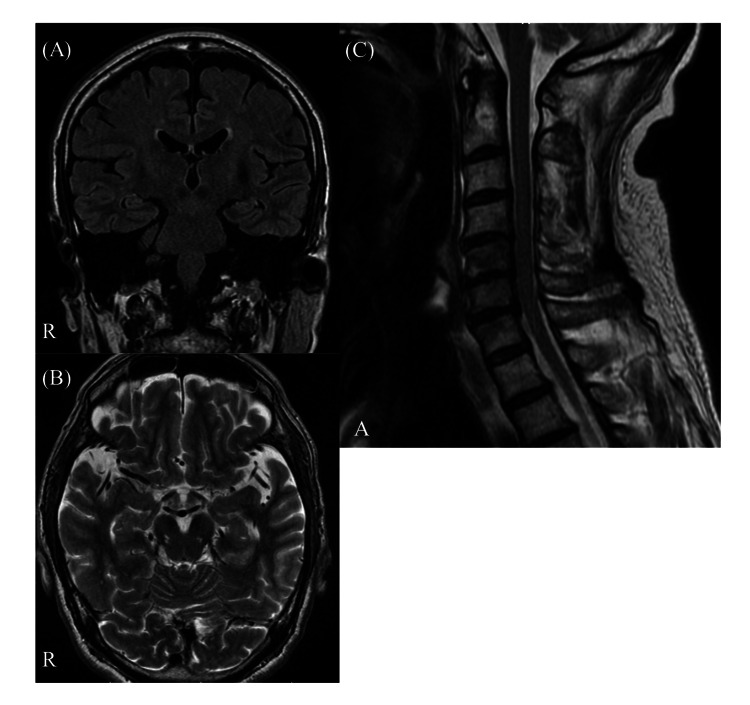
MRI of the brain (A, B) and cervical spine (C) without contrast agent on admission. (A) Coronal FLAIR MRI of the brain without contrast agent. (B) Axial T2-weighted MRI of the brain at the midbrain and hippocampus level. (C) Sagittal T2-weighted MRI of the cervical spine. All these images show no significant abnormal findings. MRI, magnetic resonance images; FLAIR, fluid-attenuated inversion-recovery

**Figure 2:**
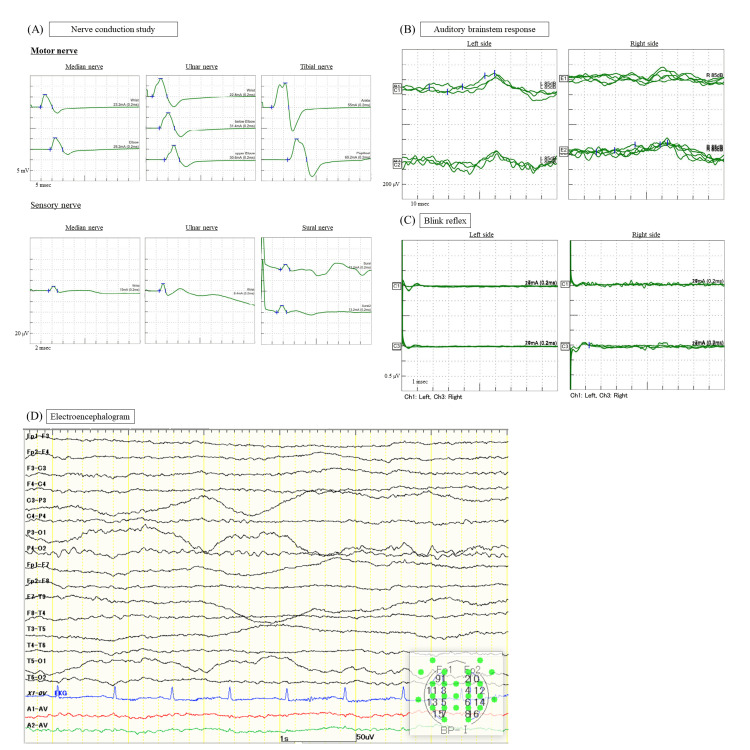
Results of neurophysiological examination. (A) NCS for median, ulnar, and tibial nerve as motor nerve and median, ulnar, and sural nerve for sensory nerve. NCS did not reveal peripheral neuropathy such as conduction block, decreased amplitude, or decreased conduction velocity. (B) ABR recorded waveforms at 85 dB nHL. The upper traces represent reflex responses of the left ear and the lower traces represent those of the right. The left figure depicts the left side stimulation and the right figure represents the right side. The responses of each side are low voltage with no prolonged latency although the right side is affected by artifact. (C) Blink reflex in response to supraorbital nerve stimulation. The upper traces represent reflex responses of the left orbicularis oculi muscles. The lower traces represent reflex responses of the right orbicularis oculi muscles. The left figure depicts the left side of supraorbital nerve stimulation and the right figure shows the right side. Both R1 and R2 reflexes are poor on each side. (D) EEG record without sedation. EEG showed a slow (7–8 Hz) and low amplitude posteriorly dominant rhythm wave, without any epileptic discharge. NCS, nerve conduction study; ABR, auditory brainstem response; EEG, electroencephalography

Blood tests showed only mildly elevated inflammatory response (9100 white blood cells/μL (normal: 4000-8500 /μL)), CRP: 1.77 mg/dL (<0.3 mg/dL)). Serum antibody titer determined by complement fixation (CF) test for *Mycoplasma pneumoniae* was 4 (<64). On cerebrospinal fluid (CSF) examination, oligoclonal bands were positive, although cell count, protein, and IgG index were within normal range. After the immunotherapy described below, the patient was found to be positive for serum anti-Gal-C IgG antibody in enzyme-linked immunosorbent assay (ELISA) method; all other measured antibodies related to autoimmune diseases, including anti-GQ 1b antibody, were negative. MRI of the brain (Figures 1A, 1B) and cervical spine (Figure 1C) and nerve conduction studies (Figure 2A) revealed no abnormalities. Evoked decreases in auditory brainstem response (ABR) (Figure 2B) and poor response on bilateral Blink reflex examination (Figure 2C) were observed. Electroencephalogram examination showed a slow (7-8 Hz) and low amplitude posteriorly dominant rhythm wave, without any epileptic discharge (Figure 2D) (Table 2).

**Table 2:** Performed electrophysiological tests and their brief result. ABR, auditory brainstem response

Electrophysiological test	Brief result
Nerve conduction study	No conduction block, decreased amplitude, or decreased conduction velocity
ABR	The responses of each side are low voltage with no prolonged latency
Blink reflex	Both R1 and R2 reflexes are poor on each side
Electroencephalography	Slow (7-8 Hz) and low amplitude posteriorly dominant rhythm wave

**Figure 3:**
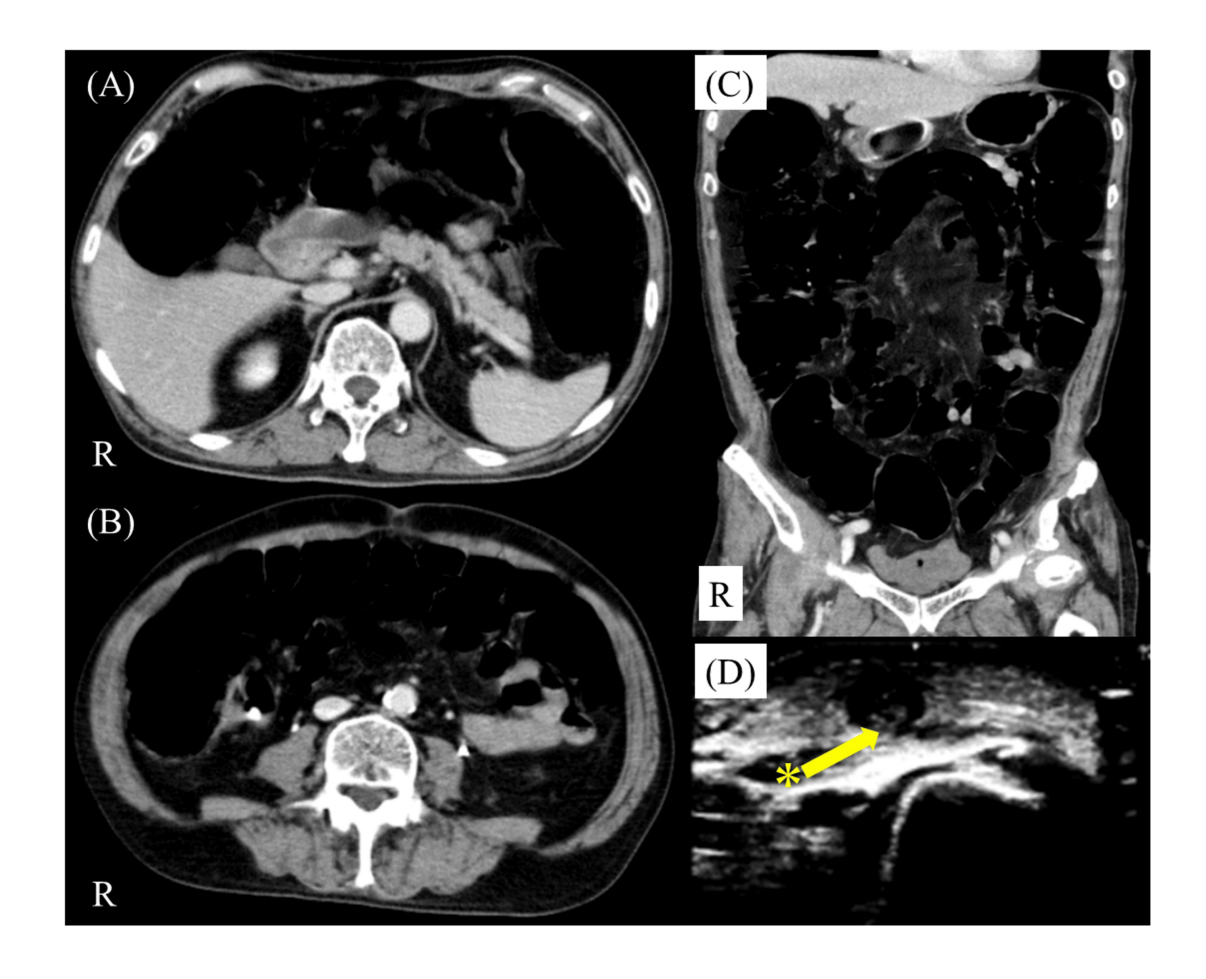
Contrast-enhanced CT scan of the abdomen (A-C) and echocardiographic image of the right forearm (D). (A) Horizontal section with cranial level. (B) Horizontal section with caudal level. (C) Coronal sections. These CT images showed gas retention and abnormal dilatation of the entire intestinal tract without obstruction. (D) A thrombus with high echogenicity was observed in the cutaneous vein (*). CT, computed tomography

Based on his drinking habits and symptomatic course, we considered Wernicke's encephalopathy and started high-dose vitamin B1 administration (1500 mg/day for three days then 250 mg/day for five days) at the day of admission; however, he did not respond to the treatment. Serum vitamin B1 was later found to be normal and the treatment was terminated. Methylprednisolone (mPSL) pulse (1 g/day for three days) was started on the eighth day of admission. On the ninth day, abdominal pain and distention appeared. Abdominal computed tomography (CT) showed gas retention and abnormal dilatation of the entire intestinal tract without obstruction, consistent with paralytic ileus (Figures 3A, 3B, 3C). On the 10th day of admission, decreased levels of consciousness to GCS E1V2M4 and SpO_2_ were observed. The patient also exhibited glossoptosis and saliva retention probably due to dysphagia; therefore, he was managed with artificial ventilation. Re-examination of the brain MRI and electroencephalography (EEG) on the next day showed no change in his condition. On the 11th to 15th day, the first course of IVIG (0.4 g/kg/day) was conducted. There was no response immediately afterward. Around one week after the start of the IVIG therapy, his respiratory condition gradually started to improve and became stable. Afterward, the patient was extubated and weaned from ventilatory management. On the 24th day, swelling and redness appeared in the right forearm. Superficial vein thrombosis was confirmed with ultrasonography (Figure 3D) and treated with heparin. No additional IVIG therapy was performed due to concerns about thrombotic side effects associated with IVIG. Two and a half months after hospitalization, he underwent a follow-up brain MRI, and there was still no unremarkable finding or change. The patient's symptoms slowly improved, and at four months, he could speak despite dysarthria and walk short distances with assistance. The serum anti-Gal-C IgG antibody became negative in the follow-up test at that time. Ultimately, we diagnosed him with anti-Gal-C antibody-associated BBE. He was transferred to a rehabilitation facility.

## Discussion

The diagnostic criteria for BBE advocate the following: 1) decreased level of consciousness, 2) bilateral external ophthalmoplegia, and 3) ataxia; these are of subacute onset and progress within four weeks [5]. Our case meets these criteria. Furthermore, abnormalities of the Blink reflex and ABR have been reported in BBE cases, and the presentation of our case was also consistent with these findings [4,6,7]. Although our case had no abnormality in the brain MRI, the frequency of abnormal findings in BBE cases is reported to be 11% [1]. The result of MRI of our patient does not rule out BBE and the utility of brain MRI is limited for making a diagnosis. However, our patient had an atypical presentation for BBE as he tested positive for anti-Gal-C antibodies, had no antecedent infection, and had a poor outcome.

Galactocerebroside is a glycolipid and a component of myelin hence anti-Gal-C antibodies, targeting this structure, induce either central or peripheral nervous system disorders [8]. Although this antibody has been reported to contribute to the development of demyelinating Guillain-Barré syndrome, this case is valuable as no previous report has described the detailed course of BBE in adults [9]. Table 3 summarizes the reports of central nervous system (CNS) diseases with anti-Gal-C antibodies [10-13].

**Table 3:** Summary of previously reported cases of anti-Gal-C-associated CNS diseases. BBE, Bickerstaff's brainstem encephalitis; Abx, antibiotics; IVMP, intravenous methylprednisolone; Gal-C, galactocerebroside; CNS, central nervous system; DEX, dexamethasone; IVIG, intravenous immunoglobulin

Literature	Number of cases	Age	Antecedent infection	Type of diseases	Treatment	Outcome
Sugeno N, et al. 2012. [10]	1	36	(+)	Meningoencephalitis	ABx (minocycline)	Remission
Samukawa M, et al. 2012. [11]	4	44-73	(+): 1/4	ADEM	IVMP+IVIG: 3, IVMP: 1	Treatment response (+): 2/4
Sauteur PM, et al. 2015. [12]	1	9	(+)	BBE	NA	NA
Kuwahara M, et al. 2017. [13]	5	4-17	(+): 4/5	Encephalitis: 1; encephalopathy: 1; brainstem encephalitis: 1; meningoencephalitis: 1; bilateral striatal necrosis: 1	IVMP+ABx: 2, DEX+ABx: 1, ABx: 2	Treatment response (+): 4/5
Our case	1	68	(-)	BBE	IVMP+IVIG	Partial response

The age of disease onset is widely distributed from infants to elderly persons. The types of disease are diverse, including encephalitis, brainstem lesions, meningoencephalitis, and acute disseminated encephalomyelitis (ADEM) [10-13]. Preceding infection was also observed in seven of 11 reported cases, suggesting a relatively high frequency. Some kind of immunotherapy (IVMP, intravenous methylprednisolone; six cases, dexamethasone (DEX); one case, IVIG; three cases, all in combination with IVMP) was conducted in the majority of patients (seven out of 11 patients). Antibiotics were also used in six cases, three of which were combined with steroid treatment as described above. Seven of the 10 described cases had efficacy for treatment although the detailed degree of improvement is unclear. The Gal-C antibody-related CNS disease is not so common but the proportion of patients who would benefit from treatment is relatively high.

It is interesting that the age of onset differs depending on the disease type. Notably, many of the patients who exhibited ADEM are older patients [11]. Additionally, these ADEM group patients tend to have a poorer response to treatment than the other patients. In this case, repeated brain and cervical MRIs showed no demyelinating lesions and ADEM is incompatible. To date, no case of BBE in older individuals has been reported. Our case had no meningeal sign or CSF pleocytosis and the complication of meningitis was not indicated.

Respiratory infection is the most common type of preceding infection of BBE and it occupies approximately 60% [1,14]. In a previous report comparing BBE cases that were classified into anti-GQ1-b antibody-positive and negative cases, the frequency of prior infection was significantly lower in the anti-GQ1-b antibody-negative cases, with approximately 50% [1]. The frequency of prior *Mycoplasma pneumoniae* infection has been reported to be significantly higher in anti-Gal-C antibody-positive GBS cases than in those without the antibody [15]. Additionally, it has been reported that anti-Gal-C IgG antibodies are positive in the CSF of a patient with GBS and additional CNS symptoms after mycoplasma infection [16]. Anti-Gal-C IgG is smaller in size than IgM and has a higher ability to cross the blood-nerve barrier of nerve roots. Hence the antibody can cause CNS lesions [17]. The present case had no clear episode of antecedent infection, and the antibody titer by the CF method did not suggest prior *Mycoplasma pneumoniae* infection. The mechanism of induction of anti-Gal-C antibody is unclear. In addition, we could not measure anti-Gal-C IgG antibodies in CSF. Nevertheless, the fact that our patient's symptoms were relieved with immunotherapy and the anti-Gal-C antibody titer became negative in the follow-up test suggests that anti-Gal-C antibody is involved in the development of BBE.

In our case, the response to immunotherapy was partial, and the outcome was relatively poor. In the comparison of anti-GQ1b antibody-positive and negative BBE cases, no clear difference in response to treatment was observed, although the number of negative cases was small [1]. Furthermore, comprehensive data on this condition are lacking. In a previous report of anti-Gal-C antibody-related CNS disease, the response to immunotherapy was poor in a group of ADEM cases [13]. Therefore, it is possible that anti-Gal-C antibody-related diseases in which the brain parenchyma, including the brainstem, is involved may show an undesirable response to immunotherapy. Our case presented complicated urinary retention and paralytic ileus. As a characteristic point of our case, autonomic dysfunctions including dysautonomia are more frequent in anti-Gal-C antibody-associated GBS than in other antibody-positive GBS [15]. Although nerve conduction studies did not suggest any peripheral motor or sensory neuropathy in this case, autonomic neuropathy may have occurred. Alternatively, BBE itself may have complicated autonomic dysfunction [1]. Additionally, our patient required artificial ventilation owing to impaired consciousness and airway problems; therefore, we had more difficulty in managing the case. Initially, we strongly suspected Wernicke's encephalopathy, and immunotherapy was delayed because inflammatory CNS diseases, including BBE, could not be considered enough. We should have conducted immunotherapy earlier in parallel with vitamin therapy. The patient’s advanced age may also have resulted in an unpreferable outcome compared to the generally recognized BBE prognosis, which is usually seen in younger patients. The evaluation of anti-Gal-C antibodies in patients with encephalitis or brainstem encephalitis with unknown causes is uncommon. Some previous patients may have been overlooked and missed the opportunities for treatment. More cases of this disease need to be accumulated in the future to establish appropriate diagnostic and therapeutic evidence.

## Conclusions

We reported a case of an older male patient with BBE, diagnosed with multiple cranial neuropathies, ataxia, impaired consciousness, and dysautonomia, and tested positive for anti-Gal-C antibody. The patient was treated with multiple immunotherapies although the response was partial and he developed complications, such as ventilatory disturbance and paralytic ileus. The prognosis of patients with anti-Gal-C antibody-positive BBE tends to be worse than that of classic BBE and treatment should be performed with caution.
